# Multicenter outcomes for ventricular assist device support for failed stage II palliation

**DOI:** 10.1016/j.jhlto.2023.100015

**Published:** 2023-11-17

**Authors:** Edon J. Rabinowitz, Mary Mehegan, Anna Joong, Muhammad Shezad, Angela Lorts, Chet R. Villa, Jennifer Conway, Ryan Kobayashi, Scott R. Auerbach, Matthew Zinn, Robert Niebler, Mehreen Iqbal, John Dykes, Swati Choudhry, Othman Aljohani, Mohammed Absi, Michelle S. Ploutz, Eric R. Griffiths, Matthew J. O’Connor, Deepa Mokshagundam, Ahmed S. Said

**Affiliations:** a Division of Pediatric Critical Care Medicine, Washington University in St Louis, St Louis, Missouri; b Division of Pediatric Cardiology, Washington University in St Louis, St Louis, Missouri; c Division of Pediatric Cardiology, Lurie Children’s Hospital, Chicago, Illinois; d The Heart Institute, Cincinnati Children’s Hospital, University of Cincinnati, Cincinnati, Ohio; e Division of Pediatric Cardiology, Stollery Children’s Hospital, Edmonton, Alberta, Canada; f Division of Pediatric Cardiology, Boston Children’s Hospital, Boston, Massachusetts; g Department of Pediatrics, Division of Cardiology, University of Colorado School of Medicine, Children’s Hospital Colorado, Aurora, Colorado; h Division of Pediatric Cardiology, Children’s Hospital of Pittsburgh, Pittsburgh, Pennsylvania; i Medical College of Wisconsin, Department of Pediatrics, Section of Critical Care, Milwaukee, Wisconsin; j Division of Pediatric Cardiology, Children’s Medical Center Dallas, Dallas, Texas; k Division of Pediatric Cardiology, Lucile Packard Children’s Hospital, Palo Alto, California; l Division of Pediatric Cardiology, Texas Children’s Hospital, Houston, Texas; m Division of Pediatric Cardiology, Benioff Children’s Hospital, San Francisco, California; n Heart Institute, Le Bonheur Children’s Hospital, University of Tennessee, Memphis, Tennessee; o Division of Pediatric Cardiology, University of Utah, Salt Lake City, Utah; p Division of Cardiothoracic Surgery, University of Utah, Salt Lake City, Utah; q Division of Pediatric Cardiology, Children’s Hospital of Philadelphia, Philadelphia, Pennsylvania

**Keywords:** ventricular assist device, stage 2 palliation, Glenn, transplantation, pediatrics, single ventricle

## Abstract

**Background:**

Ventricular assist device (VAD) use for failed stage II palliation (S2P) is increasing with limited data on outcomes.

**Methods:**

To address this knowledge gap, we conducted a multicenter retrospective review of the Advanced Cardiac Therapies Improving Outcomes Network registry. We leveraged the registry to analyze data on the clinical course, complications, and survival of systemic VADs (SVAD) after S2P.

**Results:**

We identified 34 patients from 15 centers between 2012 and 2022 implanted at median age of 1.8 years [interquartile range (IQR) 0.9-2.7]; 85% had systemic right ventricles and all patients underwent at least one sternotomy. Preimplant, all but 1 patient had an Interagency Registry for Mechanically Assisted Circulatory Support profile of 1-2, with 62% being on ≥2 inotropes, 50% total parenteral nutrition dependent, 38% mechanically ventilated, and 20% on extracorporeal membrane oxygenation in the week preceding implant. Device strategy was variable with 70% being on continuous flow devices and the remaining on pulsatile support. Multiple device strategies were utilized in 32% of patients. Median time on VAD support was 74 days [IQR 30-186]. Adverse events were frequent and included infection (47%), strokes (29%), bleeding (26%), dialysis (15%), and respiratory failure (9%). Bleeding complications (*p* = 0.0004), respiratory failure (*p* = 0.04), and multiple inotropes preimplant (*p* = 0.046) were associated with in-hospital mortality. Overall survival to transplant/recovery was seen in 76% and to 1-year postexplant in 56%.

**Conclusion:**

Encouraging clinical outcomes are seen with SVAD use for failed S2P even in the face of frequent adverse events and wide center variability in device strategy. Ongoing multi-institutional collaboration is required to better understand optimal SVAD support strategies.

## Graphical abstract

**Figure:**
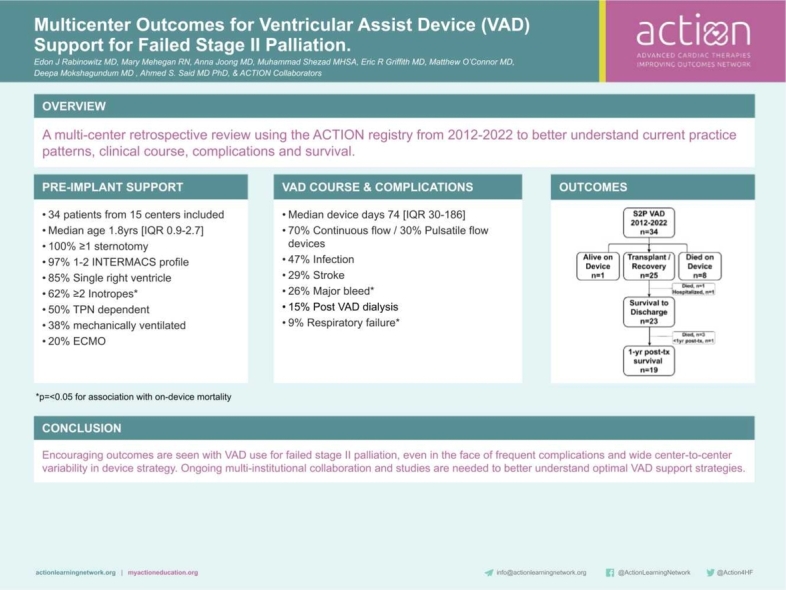

Stage II single ventricle palliation (S2P) can fail for multiple reasons, including systolic or diastolic dysfunction, anatomic obstructions, valve dysfunction, or incessant arrhythmias.^1^, ^2^, ^3^ Patients with S2P failure are especially challenging given the baseline cyanosis that may not necessarily improve with ventricular assist device (VAD) support,^2^, ^3^ and may even worsen due to dichotomous central venous return between the upper and lower body.^1^, ^2^, ^3^ Uneven decompression could worsen hypoxia with more veno-venous collateral formation from the upper to lower body. This can be compounded by the additive effect of elevated pulmonary vascular resistance (PVR), upper body systemic vascular resistance (SVR), or arteriovenous malformations (AVMs). These altered baseline physiologic conditions can exacerbate increased end-organ oxygen demand and volume load on a fragile single ventricle.

Over the past decade, reports of VAD utilization as a rescue strategy for failed S2P physiology have been increasing.^2^, ^3^, ^4^, ^5^, ^6^, ^7^, ^8^, ^9^, ^10^ While these reports expand on VAD strategies, care, and outcomes for failed S2P, data remain limited to single-center case reports and small case series. These limited reports are suggestive of wide center-to-center variation in care and timing of VAD deployment, including limited center offering of VAD for failed S2P. To address this knowledge gap, we queried the Advanced Cardiac Therapies Improving Outcomes Network (ACTION) registry, a multicenter pediatric collaborative aimed at improving outcomes in children with heart failure.^11^ Using this dataset, we sought to describe preimplant clinical characteristics, VAD implant strategies, survival outcomes at transplant, hospital discharge, and 1-year postexplant, as well as adverse events among patients supported with VAD for failed S2P.

## Materials and methods

We conducted a retrospective ACTION registry analysis of all children supported with single VADs (SVAD) from June 2012 to June 2022 with follow-up outcome data available through January 2023. Patients who were previously unoperated, underwent only stage I palliation (S1P) or proceeded to stage III palliation (S3P) prior to VAD operation were excluded. This study was approved by the Washington University in St. Louis Institutional Review Board (IRB) and the ACTION registry itself was approved by the IRB at Cincinnati Children’s Hospital where the data are housed. Data collection included clinical and surgical characteristics before and after SVAD support and adverse events (with censoring at time of SVAD explantation) as previously defined by the ACTION registry.^11^ Chart abstraction by trained registry personnel was later adjudicated for selected adverse events by 2 blinded reviewers and a committee to maintain registry definition standards. Complete study variable definitions are available for review (Supplementary digital content 1). One-year postexplant data were collected from participating centers directly using a 1-time supplemental inquiry. Competing outcomes were assessed and included mortality, transplanted, recovered patients, and those who remained on SVAD support at the time of the analysis, with censoring at time of transplantation.

Statistical analysis was performed using GraphPad version 9 (GraphPad Software, CA). Descriptive statistics, with frequencies and percentage or median and interquartile range (IQR), were used to depict patient characteristics. Predefined variables were analyzed both individually and in composite form, and compared in survivors vs nonsurvivors. Survival was assessed using Kaplan-Meier curves, with censoring at time of VAD exaplnatation. Group comparisons of subject characteristics were done with Mann-Whitney test for quantitative variables and chi-square test for categorical variables. Logistic regression models were used to analyze adverse event rates in survivors and nonsurvivors. A *p*-value of <0.05 indicated statistical significance.

## Theory

Support of the failed S2P patient with VAD has historically been uncommon, though the practice has been gaining attention in recent years. This registry-based analysis may shed light on current practice patterns and open the door for further studies, collaboration, optimal patient selection, and support strategies moving forward.

## Results

### Preimplant patient characteristics and support

At the time of this study, ACTION registry data were available from 45 centers and 34 patients from 15 centers from 2012 to 2022 met inclusion criteria (Table 1). The median age at implant was 1.8 years [IQR 0.9-2.7]. Patients weighed a median of 10 kg [IQR 8-13] and had a body surface area of 0.47m^2^ [IQR 0.4-0.56]. Most patients (85%) had systemic right ventricles and ≥2 previous sternotomies (89%). Interagency Registry for Mechanically Assisted Circulatory Support (INTERMACS) profile at implant was 1 in 29%, 2 in 68%, and 3 in 3%. A history of cerebrovascular accidents (CVA) prior to VAD implant was reported in 15%.

**Table 1:** Patient Characteristics and Preimplant Support

Variable median [IQR]	Total pts *n* = 34	In-hospital outcome		*p* value
		Survived *n* = 26	Died *n* = 8	
Age at implant, years	1.8 [0.9-2.7]	1.7 [0.8-2.7]	2.1 [1.5-2.8]	0.38
Wt at implant, kg	10 [8-13]	10 [8.1-12.5]	9.9 [8.1-13.5]	0.64
BSA, m2	0.47 [0.4-0.56]	0.47 [0.39-0.55]	0.45 [0.39-0.59]	0.78
Female, *n* (%)	12 (35%)	10 (36%)	2 (33%)	0.68
Systemic ventricle, *n* (%)	0.99			
RV	29 (85%)	22 (85%)	7 (87%)	-
LV	3 (9%)	3 (11%)	0 (0%)	-
Unspecified	2 (6%)	2 (8%)	0 (0%)	-
Prior sternotomies, *n* (%)				0.64
1	4 (11%)	4 (16%)	0 (0%)	-
2	18 (53%)	14 (54%)	4 (50%)	-
3	5 (15%)	3 (11%)	2 (33%)	-
≥4	7 (21%)	5 (19%)	2 (25%)	-
INTERMACS profile at implant, *n* (%)				0.66
1	10 (29%)	7 (27%)	3 (38%)	-
2	23 (68%)	18 (69%)	5 (62%)	-
3	1 (3%)	1 (4%)	0 (0%)	-
History of CVA, *n* (%)	5 (15%)	4 (14%)	1 (17%)	0.99
Direct Implant from ECMO, *n* (%)	7 (20%)	6 (21%)	1 (17%)	0.99
Disease Burden (in 1 week preimplant)				0.34^a^
Mechanical ventilation, *n* (%)	13 (38%)	9 (35%)	4 (50%)	0.68
NMBA, *n* (%)	3 (9%)	2 (11%)	1 (12%)	0.51
TPN dependency, *n* (%)	17 (50%)	12 (46%)	5 (63%)	0.39
Dialysis, *n* (%)	0 (0%)	0 (0%)	0 (0%)	NS
# of inotropes, *n* (%)				0.046^b^
1	9 (28%)	5 (19%)	4 (66%)	-
2	17 (53%)	15 (58%)	2 (25%)	-
3	3 (9%)	1 (4%)	2 (25%)	-
Unspecified	2 (6%)	2 (8%)	0 (0%)	-

Abbreviations: BSA, body surface area; CVA, cerebrovascular accident; ECMO, extracorporeal membrane oxygenation, INTERMACS, Interagency Registry for Mechanically Assisted Circulatory Support; LV, left ventricle; NMBA, neuromuscular blocking agent; RV, right ventricle; TPN, total parenteral nutrition; Wt, weight.

Mann-Whitney U test for continuous variables and chi-square test for categorical variables.

a Composite analysis of combined disease burden variables as related to in-hospital mortality.

b *p* < 0.05.

During the week immediately preceding VAD implantation, patients required a substantial amount of support. This included 20% directly implanted from extracorporeal membrane oxygenation (ECMO) (of which notably all but 1 patient survived to hospital discharge). Almost all patients (90%) were on inotropic support with 62% being on ≥2 inotropes at the time of implantation. Mechanical ventilation was utilized in 38% of patients, including 50% of those who died and a small number of patients (9%) were under neuromuscular blockade. Half the patients were dependent on total parenteral nutrition (TPN, 50%), with a higher percentage (63%) seen in those who died. No patient required dialysis in the week preceding implant.

### SVAD implantation strategies

**FIGURE 1:**
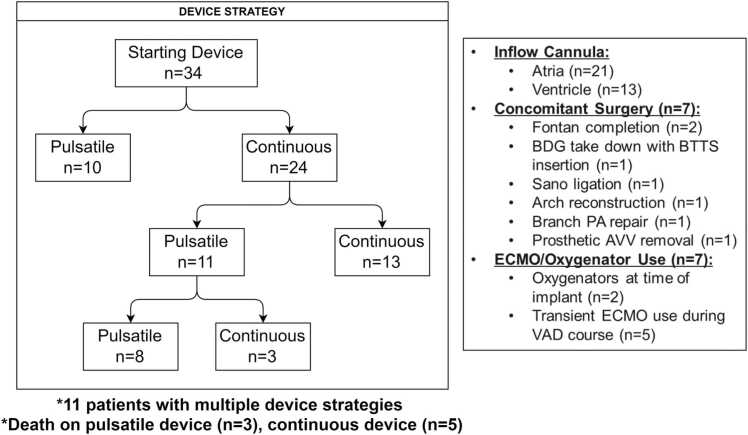
Device implant strategy. Initial device implant strategy is shown. Nearly 30% of initial devices were pulsatile, of which patients remained on same device strategy. The remaining 70% of patients were initially supported with continuous flow devices. By the time of explant, 46% of these patients had undergone multiple device strategies. Additional device cannulation strategies, concomitant procedures, and extracorporeal membrane oxygenator/oxygenator use are listed. AVV, atrioventricular valve; BDG, bidirectional Glenn; BTTS, Blalock-Thomas-Taussig shunt; ECMO, extracorporeal membrane oxygenator; PA, pulmonary artery; VAD, ventricular assist device.

Device utilization strategies were variable among centers (Figure 1). Most patients were implanted with the intention of bridging to transplantation (82%) with the remaining implanted as a bridge to candidacy (12%) or recovery (6%). Several patients had multiple device strategies employed. Of the 24 patients (71%) who were initially implanted with continuous flow devices, 13 (54%) remained on continuous flow devices and 11 (46%) transitioned to pulsatile flow devices. In total, 11 patients (46%) were supported on multiple device strategies with 3 patients (9%) undergoing 2 or more device type changes. Ten patients (29%) were initially implanted with pulsatile flow devices and remained on this device type for their entire course. Device types used are available for review (Supplemental digital content 2). A total of 62% of patients were cannulated in the atria and the remaining in the ventricle directly and only 20% of patients reported additional surgery at the time of VAD implantation. This included 2 Fontan completions and 1 conversion to aortopulmonary shunt physiology. ECMO or oxygenator use was employed in 7 patients (20%) during the VAD course. Two of the described oxygenators were utilized in the immediate postimplant period as acute postoperative recovery strategies and the remaining were utilized at a later pre-explant date.

### SVAD course and outcomes

**FIGURE 2:**
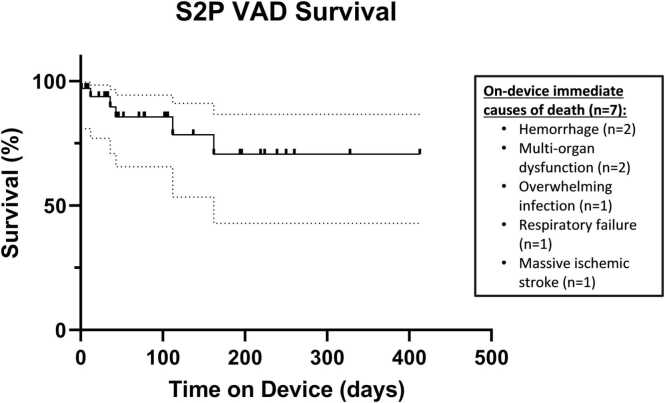
Stage II single ventricle assist device on-device survival. Kaplan Meier curve of on-device survival to transplant. On-device causes of death are listed. S2P, stage II palliation; VAD, ventricular assist device.

The study cohort remained on SVAD support for a median of 74 days [IQR 30-186], while those who died spent a relatively shorter median of 40 days on-device [IQR 15-149] (*p* = 0.44). Over one-third (38%) of deaths occurred in the first 4 weeks postimplant. Overall, on-device survival to transplant or recovery was 76% (Figure 2). Causes of death while on support included major bleeding (*n* = 2), multiorgan dysfunction (*n* = 2), sepsis (*n* = 1), respiratory failure (*n* = 1), ischemic CVA (*n* = 1), and unknown (*n* = 1). On-device mortality occurred on both continuous (*n* = 5) and pulsatile (*n* = 3) devices (*p* = 0.43) and included 1 of the 2 reported Fontan completion patients, and the single reported aortopulmonary shunt conversion patient.

There were a total of 81 reported adverse events in 25 patients (74%). These occurred in most survivors (65%) and in all patients who died on-device (100%, *p* = 0.08). Adverse events (Table 2) included 11 central nervous system (CNS) injuries in 10 patients (29%): ischemic CVA (*n* = 8), hemorrhagic CVA (*n* = 1), transient ischemic attack (*n* = 1), and intracranial bleed (*n* = 1). CNS events occurred a median of 26 days postimplant [IQR 5-68] with 30% of events occurring in the first postimplant week. Other adverse events included 15 major bleeds in 9 patients (27%) and occurred at higher rates in those who died (75%, *p* = 0.0004). Note that 3 major bleeding events occurred within 24 hours of implant, and the remaining >1 week. A total of 27 infections were seen in 11 patients (32%) with blood stream infections consisting of half reported events. Dialysis was used in 5 patients (15%) and disproportionally in those who died (60%, *p* = 0.06). Respiratory failure was reported in 2 patients (6%) and both died on device (*p* = 0.04). Device malfunction was reported 7 times in 4 patients (12%) and occurred mostly with Centrimag use (86%).

**Table 2:** On-Device Complications

Complications by # of occurrences (*n* of pts, %) Median [IQR]	Total pts *n* = 34	In-hospital outcome		*p* value
		Survived *n* = 26	Died *n* = 8	
Device days	74 [30-186]	90 [32-202]	40 [15-149]	0.44
Total # of adverse events	81 (25, 74%)	48 (17, 65%)	33 (8, 100%)	0.08
Immediate event outcome, *n* (%)^a^				
Listing removal	5 (15%)	3 (5%)	2 (33%)	0.34
Temporary	5 (15%)	3 (5%)	2 (33%)	-
Permanent	0 (0%)	0 (0, 0%)	0 (0, 0%)	-
ECMO	2 (6%)	0 (0%)	2 (25%)	0.009^b^
Invasive intervention	7 (20%)	5 (19%)	2 (25%)	0.72
Death	4 (12%)	0 (%)	4 (50%)	-
CNS events	11 (10, 29%)	8 (7, 27%)	3 (3, 37%)	0.32
Ischemic CVA	8 (8, 23%)	6 (6, 23%)	2 (2, 25%)	0.22
Hemorrhagic CVA	1 (1, 3%)	0 (0, 0%)	1 (1, 12%)	0.23
TIA	1 (1, 3%)	1 (1, 4%)	0 (0, 0%)	0.99
Intracranial bleed	1 (1, 3%)	1 (1, 4%)	0 (0, 0%)	0.99
Days, implant to CNS event	26 [5-68]	13 [5-58]	43 [25-63]	0.52
Major bleeds	15 (9, 26%)	3 (3, 12%)	12 (6, 75%)	0.0004^b^
Post-op bleed	5 (3, 9%)	0 (0, 0%)	5 (3, 37%)	0.001
Central line insertion site	3 (1, 3%)	0 (0, 0%)	3 (1, 12%)	0.07
Dialysis related	1 (1, 3%)	0 (0, 0%)	1 (1, 12%)	0.23
GI related	3 (3, 9%)	2 (2, 8%)	1 (1, 12%)	0.67
Airway related	1 (1, 3%)	1 (1, 4%)	0 (0, 0%)	0.99
Unspecified	2 (2, 9%)	0 (0, 0%)	2 (2, 25%)	-
# of events, timing of major bleed				
≤1 week postimplant	3 (3, 20%)	0 (0, 0%)	3 (3, 25%)	0.99
>1 week postimplant	12 (6, 80%)	3 (3, 25%)	9 (3, 75%)	0.99
Major infection	27 (16, 47%)	19 (13, 50%)	8 (5, 62%)	0.69
Blood stream, (*n* of pts, %)	11 (8, 23%)	8 (5, 19%)	3, (3, 37%)	0.28
Mediastinitis, (*n* of pts, %)	2 (2, 9%)	2 (2, 8%)	0 (0, 0%)	0.42
Cannula site infection	4 (4, 12%)	4 (4, %)	0 (0, 0%)	0.24
NEC	3 (2, 9%)	3 (2, 8%)	0 (0, 0%)	0.42
Pneumonia	2 (2, 9%)	2 (2, 8%)	0 (0, 0%)	0.42
*Clostridium difficile*	1 (1, 3%)	0 (0, 0%)	1 (1, 12%)	0.23
Dialysis occurrences	5 (5, 15%)	2 (2, 8%)	3 (3, 37%)	0.06
Respiratory failure	2 (2, 9%)	0 (0, 0%)	2 (2, 25%)	0.04^b^
Pericardial effusion	1 (1, 3%)	1 (1, 4%)	0 (0, 0%)	0.99
Arrhythmia	1 (1, 3%)	0 (0, 0%)	1 (1, 12%)	0.23
DVT	2 (2, 9%)	2 (2, 8%)	0 (0, 0%)	0.42
Hemolysis	9 (9, 26%)	7 (7, 27%)	2 (2, 25%)	0.91
Psychiatric event	1 (1, 3%)	1 (1, 4%)	0 (0, 0%)	0.57
Device malfunction	7 (4, 20%)	7 (7, 27%)	0 (0, 0%)	0.10
Centrimag	6 (3, 9%)	6 (6, 23%)	0 (0, 0%)	-
Berlin Heart	1 (1, 3%)	1 (1, 4%)	0 (0, 0%)	-

Abbreviations: CNS, central nervous system; CVA, cerebrovascular accident; DVT, deep vein thrombosis; ECMO, extracorporeal membrane oxygenation; GI, gastrointestinal; NEC, necrotizing enterocolitis; NS, nonsignificant; TIA, transient ischemic attack.

Mann-Whitney U test for continuous variables, chi-square test for categorical variables, and logistic regression for event rate comparisons.

a Immediate event outcomes available in only 58% of reported adverse events.

b *p* < 0.05.

Of the total adverse events reported, 58% also included documentation of immediate interventions required (e.g., blood product transfusion or initiation of antibiotics). Of note, 38% of adverse events were reported to have directly resulted in conversions to ECMO, invasive interventions (e.g., unanticipated catheterization or surgical intervention) or patient demise (Table 2). Two of the 7 ECMO/oxygenator patients (28%) died on-device (*p* = 0.57).

**FIGURE 3:**
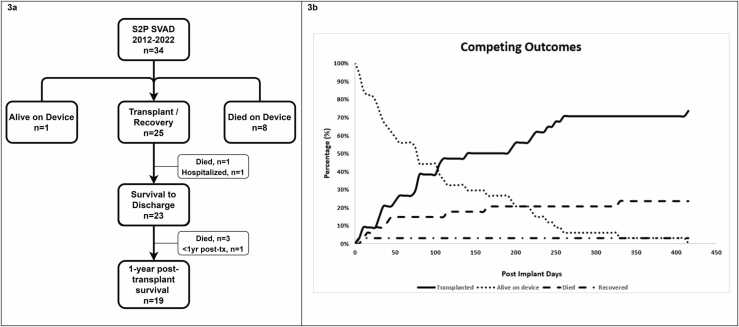
Short- and long-term outcomes of single ventricular assist device following stage 2 palliation. (a) At the time of this study, 25/34 (74%) device supported patients were successfully bridged to either transplant (*n* = 24) or recovery (*n* = 1). One additional patient was alive on device (3%) and 8 patients (24%) died on device. Postexplant outcomes included 23 (68%) patients who were successfully discharged and 19 (56%) patients who were alive at 1-year postexplant. Note that 2 patients remained hospitalized at the time of this study and that 1 was discharged and alive <1-year post-transplant. (b) Competing outcomes depicting mortality, transplanted, recovered patients, and those who remained on device support at the time of the analysis. S2P, stage 2 palliation; SVAD, single ventricular assist device; Tx, transplant.

Of the 25/34 patients (76%) who survived to transplant (*n* = 24) or recovered (*n* = 1), 23 (68%) survived to hospital discharge (note that 1 patient remains hospitalized post-transplant), and 19 (56%) are alive 1-year postexplant (note that 1 patient is alive <1-year postexplant, Figure 3a, b). Those who were discharged (*n* = 23) had a 90% survival rate 1-year postexplant. For the most part, there were no adverse events either independently or in composite analysis that were associated with mortality. The exceptions to this were that statistically significant differences were seen in the occurrences of major bleeds or respiratory failure in those who died compared to survivors; these are unlikely clinically impactful given the small sample size. None of the SVAD implantation variables including, device types, multiple device utilization, chamber of cannulation, ECMO/oxygenator use, or additional surgery, were statistically associated with mortality. None of the preimplant characteristics had a statistically significant association with mortality either individually or in composite analysis except for a statistically significant difference in the number of inotropes used pre-SVAD in survivors compared to those who died (*p* = 0.04).

### SVAD hematologic strategies

Antithrombotic strategy evolved during the study period with increased use of bivalirudin after 2018 especially, in line with ACTION anticoagulation harmonization efforts.^12^ Antithrombotic approaches were otherwise variable among centers with a range of anticoagulant and antiplatelet strategies reported (Table 3). There was no association between either mortality, occurrence of CNS event, or occurrence of major bleed and antithrombotic strategy.

**Table 3:** Antithrombotic Strategy

Antithrombotic agent		Number of patients per antithrombotic agent					
	Total pts *n* = 34	No CNS event *n* = 24	CNS event *n* = 10	*p* value	Survived *n* = 26	Died *n* = 8	*p* value
Anticoagulant							
Bivalirudin	28 (82%)	20 (83%)	8 (80%)	0.81	23 (88%)	5 (62%)	0.09
UFH	8 (24%)	6 (25%)	2 (20%)	0.75	6 (23%)	2 (25%)	0.91
LMWH	5 (15%)	2 (8%)	3 (30%)	0.10	2 (8%)	3 (38%)	0.07
Warfarin	3 (9%)	2 (8%)	1 (10%)	0.99	3 (11%)	0 (0%)	0.31
Argatroban	0 (0%)	0 (0%)	0 (0%)	-	0 (0%)	0 (0%)	-
Antiplatelet							
ASA	25 (74%)	17 (71%)	8 (80%)	0.58	18 (69%)	7 (87%)	0.31
Dipyridamole	11 (32%)	6 (25%)	5 (50%)	0.15	9 (35%)	2 (25%)	0.61
Clopidegrol	11 (32%)	8 (33%)	3 (30%)	0.84	8 (31%)	3 (38%)	0.72
Cangrelor	1 (3%)	0 (0%)	0 (0%)	-	1 (4%)	0 (0%)	0.99

Abbreviations: ASA, acetylsalicylic acid; LMWH, low molecular weight heparin; UFH: unfractionated heparin.

Mann-Whitney U test for continuous variables and chi-square test for categorical variables.

*p* < 0.05.

## Discussion

This report encompasses the largest analysis of VAD utilization for failed S2P. It is the first multicentered analysis to focus on this unique population and sheds light on current practice trends and VAD experiences for this challenging patient population. The key findings of this study are that despite the distinct physiology, severity of preimplant illness, and abundance of adverse events, 76% of patients survived to transplant or recovery, and 68% survived to hospital discharge. Further, of those discharged, 1-year survival was excellent. These findings are more encouraging than most previous reports.^2^, ^3^, ^4^, ^5^, ^6^, ^7^, ^8^, ^9^, ^10^

The most recent Pedimacs registry report of VAD implants for patients with congenital heart disease (CHD) reveals significantly worse prognosis for patients following S2P compared to non-CHD, as well as other forms of CHD including VAD implant after S3P.^9^ A 47% survival rate to transplant was described in 21 patients; most deaths occurred in those <7 kg and early postimplant, especially in the first week and mostly within the first month. This pattern of early demise was not as strongly pronounced in this ACTION registry analysis.

Several forms of mechanical circulatory support (MCS) have been described after S2P. Initial single center descriptions of ECMO support following S2P suggested poor outcomes at best approaching ∼40% survival to discharge and high incidence of neurologic injury by Extracorporeal Life Support Organization registry analysis.^13^, ^14^, ^15^ More promising results were published from the EXCOR Investigational Device Exemption study database which included 12 patients following S2P with Berlin Heart support (Berlin Heart EXCOR, Berlin, Germany) of which 58% were bridged to transplant.^10^ This report suggested that VAD support may be a more attractive strategy than ECMO. However, more granular single center descriptions of pulsatile devices in this patient population later described complex and intense hospital courses including need for later conversions to ECMO (both on-VAD and post-transplant), hemorrhage, disseminated infections, and multiple CVAs.^3^, ^6^, ^8^

More recently there have been improved single center outcomes described with the use of continuous flow devices.^2^, ^3^, ^4^, ^7^ Though short duration of support is noted in some of the reports, Moon et al have reported their experience after S2P describing successful bridging to transplant in 9 of 10 patient with a variety of device strategies including HVAD (Medtronic, MN), Rotaflow (Getinge, Gothenburg, Sweden), and Jarvik (Jarvik Heart Inc, NY).^2^, ^3^ Interestingly, those with severe systolic dysfunction were considered for Fontan completion at the time of VAD placement; this occurred in 5 of the 10 reported patients and conversion to aortopulmonary shunt physiology is not pursued. Given the superior survival outcomes following S3P by multiple previous registry reports,^9^, ^16^ the concept of “mechanically assisted Fontan completion”^5^, ^17^ warrants further serious consideration and study. Another novel strategy for mechanical support in single ventricle physiology, including after S2P, has been described with use of total artificial heart in patients <10 kg.^18^ However reported experience remains limited having been used as salvage therapy in a single patient after a prolonged ECMO run and requires further investigation.

It has been suggested that the challenging nature of supporting S2P physiology with MCS stems from the dichotomous nature of systemic venous return.^2^, ^3^, ^5^, ^17^, ^19^ The indirect decompression of the Glenn circuit may not match the direct decompression within the cardiac chambers. With direct VAD decompression being limited to the lower body, upper and lower body systemic venous pressure differential may be exacerbated and result in veno-venous collateralization. Baseline preimplant cyanosis can therefore not only persist, but even worsen, compromising end-organ oxygen delivery despite achieving adequate cardiac output. Elevated pulmonary vascular resistance, , upper body systemic vascular resistance, and/or pre-existing arteriovenous malformations may further deepen hypoxemia, worsen end-organ oxygen delivery, and volume load on a fragile single ventricle. Over time this can potentially lead to higher VAD decompression requirements and inability to meet cardiac output demands. The analyzed ACTION data from this study lacked granularity about oxygenation or collateral burden as well as etiology pertaining to oxygenator use.

Though this study is more encouraging with regards to patient outcomes, pre and postimplant patient support to achieve a successful outcome was substantial. Almost all patients preimplant had an INTERMACS profile of 1 or 2 with 20% having been implanted directly from ECMO. ECMO use has historically been reported as an ineffective way to support patients with S2P physiology,^13^, ^14^, ^15^ however this data raise the possibility that it may be an effective strategy for acute recovery to allow for conversion to more durable forms of MCS as all but 1 preimplant ECMO supported patient survived to transplant. A statistically significant difference was seen in the number of inotropes preimplant between survivors and those who died. But it is difficult to place much weight in this finding due to the descriptive nature of this study.

Device type and implant strategies were variable in the study cohort. Center-to-center variability in decision making with regards to timing and strategy of VAD implantation has been reported^20^ and so it is not surprising that there was wide variability in this particular high-risk subset. It has been suggested that continuous flow devices more easily achieve ventricular unloading and meet sufficiently higher cardiac index demands.^21^ However, the data from this current report do not strongly favor one device strategy over another. It is interesting to note that those patients who started on pulsatile devices, remained on pulsatile devices throughout their VAD course. More recent multicentered quality improvement reports describe both mitigation of hematologic risk^12^ and a nonstatistically significant improvement in on-device survival in failed S1P physiology with use of pulsatile devices compared to continuous flow devices.^22^

Adverse events were reported in the majority of patients and all of those who died on-device. These complication rates were higher than previously reported for biventricular disease and similar to those reports for SVAD support for failed S1P physiology.^22^, ^23^, ^24^ CVA occurrence remained high in this study cohort similar to previous reports of S2P support,^3^, ^6^, ^8^, ^13^, ^14^, ^15^ and similar to those reported by previous registry reports for patients with CHD^9^ and S1P.^22^ It is possible that chronic hypoxemia is also directly related to the high rates of strokes as it can lead to inappropriate vasoconstriction and vascular stasis on the micro and macrovascular level in combination with endothelial dysfunction and red blood cell deformation.^25^, ^26^, ^27^, ^28^, ^29^ Perhaps this chronically hypoxemic cohort would benefit from a more aggressive and individualized antithrombotic strategy to mitigate this risk. This would have to be balanced with the frequency of major bleeding events that were seen at higher rates in those who died compared to those who survived in this study. Note that the definition of major bleeding in the ACTION registry may be too widely inclusive, incorporating in its definition any transfusion in the context of a suspected bleed beyond 1 week postimplant, and so this study may have overestimated actual clinical bleeding.

This study was limited by its heterogeneous and modest sample size as well as the quality of registry data. Despite the ACTION adjudication process, it is possible that not all relevant events to patient outcomes were either entered by participating centers or captured by the current data fields. Granular data, such as catheterizations interventions, elective ECMO cannulation vs extracorporeal cardiopulmonary resuscitation, extent of renal insufficiency (other than need for dialysis), or details regarding surgical procedures performed prior to implantation, are unavailable in the registry or not fully captured in the database. Detailed heart transplantation data were also unavailable in the registry and may have confounded post-transplant outcomes. Surgical data may have been underappreciated in the registry because based on published case series from contributing centers we expected more conversions to either aortopulmonary shunt physiology or Fontan at the time of implant. It remains important to assess these concomitant surgical strategies at the time of implant moving forward. Additionally, detailed data regarding timing of intensive therapy cessation, for example, length of mechanical ventilation or TPN dependency post-VAD implant, were unavailable to analyze as a surrogate of effective VAD support. The study was descriptive in nature, lacking comparison control group, and not powered for meaningful univariable or multivariable analyses. The associations seen with mortality in the study may not represent strong signals in this small sample size and require further investigation. It is interesting to note that the median patient cohort in this study was 1.8 years of age, relatively older than the typical S2P timing. Unfortunately, data on timing of S2P failure in relation to implant were unavailable and we cannot comment on either the chronicity of heart failure, critical illness or the timing of onset of symptoms leading to hospitalization. Pre-S2P hemodynamic catheterizations would be interesting to evaluate for concerning patterns for failed physiology; for example, diastolic dysfunction, PVR elevation. Perhaps there would be an opportunity to consider transplant candidacy before even pursuing S2P and avoid additional sternotomy or rapid deterioration post-S2P. These factors highlight the importance of further research to identify candidates that would most benefit from VAD implantation as well as the timing of such a support mechanism.

## Conclusions

VAD utilization for failed S2P remains limited in practice, though is slowly growing in select centers. This unique patient population has frequent and severe preimplant comorbidities. There is significant variation in device types and surgical strategies employed by VAD centers. Adverse events in this high-risk population are high yet 76% of patients in our cohort experienced a positive clinical outcome (transplant or recovery). Further, of those discharged, 1-year postexplant survival was excellent. This data demonstrate the ability to successfully support this unique patient population despite adversities. Individualized consideration and further multicentered investigation are required as current best practice standards and consensus regarding VAD deployment and strategy are lacking.

## Disclosure statement

The authors would like to disclose the following financial disclosures and relationships:

• Eric R. Griffiths, MD, has provided lectures for Berlin Heart at their training meetings, for which he received travel reimbursement. No honoraria were received for these activities.
• Jennifer Conway, MD, MSc, acknowledges receiving an unrestricted education grant from Abbott, which did not influence the content of this manuscript.
• Chet R. Villa, MD, has served as a previous consultant for ptc therapeutics and is currently a consultant for capricor therapeutics. However, these consulting relationships are not relevant to the content of this manuscript, which focuses on VAD support.
• Scott R. Auerbach, MD, is a member of the Clinical Events Committee for the Berlin Heart-sponsored Active Driver Trial. His involvement in this committee did not impact the content of this manuscript.
• Robert Niebler, MD, has received travel funds from Berlin Heart EXCOR to attend an annual user’s group meeting.
• ACTION receives funding support from Berlin Heart EXCOR (Berlin, Germany), Abbott Laboratories (Chicago, IL), Medtronic (Minneapolis, MN), Abiomed (Danvers, MA), Syncardia (Tucson, AZ), Bayer (Leverkusen, Germany)

These financial disclosures and relationships are provided for transparency and to ensure full disclosure of potential conflicts of interest. The authors assert that none of these relationships influenced the design, execution, or interpretation of the research presented in this manuscript.

This research did not receive any specific grant from funding agencies in the public, commercial, or not-for-profit sectors.
