# Supplementary material for: Multicenter outcomes for ventricular assist device support for failed stage II palliation
Source: JHLT Open. 2023 Nov 17;3:100015. doi: 10.1016/j.jhlto.2023.100015 (PMC11935379; doi:10.1016/j.jhlto.2023.100015)
Supplement: Supplementary file 1 — Supplementary material [file mmc1.pdf]

Advanced Cardiac Therapies Improving Outcomes Network
to Improve the Health of Pediatric and Congenital Heart Disease
Patients with Heart Failure

USER INSTRUCTIONS
FOR ACTION REGISTRY DATABASE
CLINICAL DISCOVERY PLATFORM (CDP)

ADVERSE EVENT DEFINITIONS EXCERPT

9 ADVERSE EVENT DEFINITIONS

Major Bleeding

An episode of SUSPECTED INTERNAL OR EXTERNAL BLEEDING that results in one or more of the following:

a. Death,
b. Re-operation,
c. Hospitalization,
d. Transfusion of red blood cells as follows:

If transfusion is selected, then apply the following rules:

During first 7 days post implant:

- ≥ 50 kg: ≥ 4U packed red blood cells (PRBC) within any 24-hour.
- < 50 kg: ≥ 20 cc/kg packed red blood cells (PRBC) within any 24-hour.

After 7 days post implant

- A transfusion of packed red blood cells (PRBC) after 7 days

Note: Hemorrhagic stroke is considered a neurological event and not as a separate bleeding event.

<u>Action Registry Specific considerations:</u>
A separate sub-form will be accrued and should be completed if Major Bleeding is selected as an adverse event and the following data elements should be collected:

1. Need for transfusion
2. Need for surgical intervention
3. Source of bleeding: If bleeding was not localized to the GI tract, abdominal cavity, or thoracic cavity; source should be documented as “other” and a detailed description of the bleeding location should be entered.

- Laboratory test results: Values should be those available at the time (within 48 hours) of the adverse event, and any unavailable lab values should be left blank.
    - As all anticoagulant lab data may vary based upon medical therapy used for systemic anticoagulation, antithrombotic agents used should be denoted at the time of the entered lab data.

- Collected lab data is as follows:

Laboratory Value	Unit of Measure
Hemoglobin	g/dL
Platelet Count	K/uL
Antithrombin III	%
Fibrinogen	mg/dL
Anti-factor XA	u/mL
PTT	seconds
INR	(none)

Neurological Dysfunction

Select one of the neurological dysfunction categories as defined by the neurology consult.

Transient ischemic attack:
Transient ischemic attack, defined as an acute transient neurologic deficit conforming anatomically to arterial distribution cerebral ischemia, which resolves in < 24 hours and is associated with no infarction on brain imaging (head CT performed >24 hours after symptom onset; or MRI*).

Action Registry Specific considerations:
TIA symptoms that resolve in 24 hours with no follow-up head CT, are assumed to be a TIA and only a TIA.

Ischemic stroke:
Ischemic stroke, defined as a new acute neurologic deficit (or acute encephalopathy or seizures in children <6 months**) of any duration associated with acute infarction on imaging corresponding anatomically to the clinical deficit. Ischemic stroke should be sub classified as due to arterial-distribution ischemia or due to venous thrombosis.

Ischemic stroke with hemorrhagic conversion:
This will be check in follow-up if the patient had a confirmed ischemic stroke and subsequently went on to hemorrhagic transformation or if initial presentation is a hemorrhagic stroke that can be attributed to an initial ischemic stroke

Hemorrhagic stroke (Acute symptomatic intracranial hemorrhage):
Acute symptomatic intracranial hemorrhage, defined as new acute neurologic deficit (or acute encephalopathy or seizures in children < 6 months**) attributable to Intracranial hemorrhage (ICH). ICH subtype should be specified as one or a combination of the following types: subarachnoid, intraventricular and parenchymal.

Please note that subdural hematoma should not be classified as hemorrhagic stroke, but rather as an extra-axial hemorrhage.

Extra-axial Hemorrhage (includes subdural hematoma)
Bleeding that occurs inside of the skull but outside of the brain parenchymal. The event may be symptomatic or asymptomatic.

Non-symptomatic stroke
Non-symptomatic stroke defined as a clinically covert ischemic stroke or ICH: infarction or ICH seen by surveillance imaging, without clinical findings of stroke or ICH at the time of event recognition)

Hypoxic-ischemic encephalopathy:
Acute new encephalopathy*** due to hypoxic-ischemic injury (HIE), manifest as clinically-evident signs or symptoms, or subclinical electrographic seizures found by complete neurological diagnostic evaluation to be attributable to acute global or focal hypoxic or ischemic brain injury not meeting one of ischemic stroke or ICH events as defined above.

Acute new encephalopathy:
Acute new encephalopathy*** due to other causes, manifest as clinically-evident signs or symptoms or subclinical electrographic seizures found by complete neurological diagnostic evaluation to be attributable causes other than stroke, ICH or HIE, as defined above. This category of "other" acute encephalopathy includes neurologic signs or symptoms or subclinical seizures found to be attributable to other conditions such as meningitis, toxic-metabolic or drug-related processes

*** Acute encephalopathy is a sign or symptom of some underlying cerebral disorder, and is manifest as depressed consciousness with or without any associated new global or multifocal neurologic deficits in cranial nerve, motor, sensory, reflexes and cerebellar function

Action Registry Specific considerations:
A separate sub-form will be accrued and should be completed if an ischemic, hemorrhagic, extra-axial, non-symptomatic, hypoxic or encephalopathy neurological dysfunction is selected as an adverse event. The following items should be collected:

1. Symptoms: Symptoms at the time of presentation should be entered
2. Factors present within 24 hours of the event
5. Stroke Severity after stabilization: record symptoms after 72 hours of the event

- Laboratory test results: Values should be those available at the time (within 48 hours) of the adverse event, and any unavailable lab values should be left blank.
  - As all anticoagulant lab data may vary based upon medical therapy used for systemic anticoagulation, antithrombotic agents used should be denoted at the time of the entered lab data.
  - Collected lab data is as follows:

Laboratory Value	Unit of Measure
WBC	K/uL
CRP (C Reactive Protein)	mg/dL
Hemoglobin	g/dL
Platelet Count	K/uL
Antithrombin III	%
Fibrinogen	mg/dL
Anti-factor XA	u/mL
PTT	seconds
INR	(none)

PSOM (pediatric stroke outcomes measure) short neuro exam form should be completed (if the evaluation was performed) at the time of neuro event and other time points if patient had a neurological adverse event and follow-up PSOM was performed.

Device Malfunction

A Device Malfunction occurs when any component of the MCSD system ceases to operate to its designed performance specifications or otherwise fails to perform as intended. Performance specifications include all claims made in the Instructions for Use.

Only captures major device malfunction defined as follows:

Major device malfunction, otherwise known as failure, occurs when of one or more of the components of the MCSD system either directly causes or could potentially induce a state of inadequate circulatory support (low cardiac output state) or death. A failure that was iatrogenic or recipient-induced will be classified as an Iatrogenic/Recipient-Induced Failure. A device malfunction or failure is considered major when one of the following conditions occurs:

a. Suspected or confirmed pump thrombus (in an intracorporeal pump)
b. Urgent transplantation (immediate 1A listing for transplant)
c. Pump replacement
d. Pump explant
e. Breach of integrity of drive line that required repair
f. Death

DO NOT include isolated paracorporeal pump exchanges due to device malfunction unless thrombus impaired the ability of the pump to provide blood flow.

Minor device malfunction defined as follows:

Minor device malfunction includes inadequately functioning external components which require repair or replacement but do not result in a-f mentioned above. Device malfunction does not apply to "routine" maintenance which includes repair/replacement of: external controller, pneumatic drive unit, electric power supplies, batteries and interconnecting cables.

Action Registry Specific considerations:
A separate sub-form will be accrued and should be completed if a device malfunction is selected as an adverse event. Further details regarding the type, treatment and outcome of the malfunction will be collected.

Major Infection

A clinical infection accompanied by pain, fever, drainage and/or leukocytosis that is treated by anti-microbial agents (non-prophylactic). A positive culture from the infected site or organ should be present unless strong clinical evidence indicates the need for treatment despite negative cultures. The general categories of infection are listed below:

Localized Non-Device Infection
Infection localized to any organ system or region (e.g. mediastinitis) without evidence of systemic involvement (See sepsis definition), ascertained by standard clinical methods and either associated with evidence of bacterial, viral, fungal or protozoal infection, and/or requiring empirical treatment.

Percutaneous Site and/or Pocket Infection
A positive culture from the skin and/or tissue surrounding the drive line or from the tissue surrounding the external housing of a pump implanted within the body, coupled with the need to treat with antimicrobial therapy when there is clinical evidence of infection such as pain, fever, drainage, or leukocytosis.

Internal Pump Component, Inflow or Outflow Tract Infection
Infection of blood-contacting surfaces of the LVAD documented by positive site culture or persistent bacteremia in the absence of sepsis. (There should be a separate data field for paracorporeal pump that describes infection at the percutaneous cannula site, e.g. Thoratec PVAD).

Sepsis/Blood-borne infection
Evidence of systemic involvement by infection, manifested by positive blood cultures and/or hypotension.

Mediastinitis
Evidence of infection within 4 weeks of surgery with a clinical pattern of fever, leukocytosis, and sternal instability and/or sternal wound drainage

<u>Action Registry Specific considerations:</u>
Resolution date should be documented when the antibiotic for the event was discontinued.

Cardiac Arrhythmia

Any documented arrhythmia that results in clinical compromise (e.g., abnormal VAD function [e.g., diminished VAD flow or suction events], oliguria, pre-syncope or syncope, angina, dyspnea), or requires hospitalization or treatment (drug therapy, defibrillation, cardioversion, ICD therapy (e.g., shock or anti-tachycardia pacing) or arrhythmia ablation procedure). Cardiac arrhythmias are classified as 1 of 2 types:

- Sustained ventricular tachycardia: VT resulting in clinical compromise or hospitalization that requires drug treatment, defibrillation, cardioversion, ICD therapy, or ablation procedure.
- Sustained supraventricular arrhythmia: SVT resulting in clinical compromise or hospitalization that requires drug treatment, cardioversion, ICD therapy, or arrhythmia ablation procedure.

Action Registry Special considerations:
Drug treatment in an outpatient setting that does not meet criteria are not collected.

Pericardial Effusion

Accumulation of fluid or clot in the pericardial space that requires surgical intervention or percutaneous catheter drainage. This event should be classified into those with clinical signs of tamponade (e.g. increased central venous pressure and decreased cardiac/VAD output) and those without signs of tamponade.

Action Registry Special considerations:
Method of drainage (item 6 on AE page): surgical intervention, chest tube or pericardial tap

Hemolysis

Defined as an increase in LDH >2.5 fold the upper limit of normal especially in the setting of any of the following:

- Pump malfunction and/or abnormal pump parameters
- Acute decline in hemoglobin or hematocrit
- Acute increase in total bilirubin without evidence of hepatic dysfunction
- Hemoglobinuria

Action Registry Special considerations:
For each hemolysis adverse event (AE), the outcome of the onset of hemolysis (item 7 on AE page) should be collected: Change in device settings, exchange for a different device, device replacement (same device type), or change in antithrombotic therapy.

If renal dysfunction occurs as the result of a hemolysis AE, the "Renal Dysfunction Detail" section (items 8-9 on AE page) should be completed.

Hepatic Dysfunction

An increase in any two of the following hepatic laboratory values (total bilirubin, aspartate aminotransferase/AST and alanine aminotransferase/ALT) to a level greater than three times the upper limit of normal for the hospital, beyond 14 days post-implant (or if hepatic dysfunction is the primary cause of death).

Hypertension

Defined as systolic, diastolic, or mean blood pressure greater than the 95th percentile for age (as described in the most recent NHLBI definitions of hypertension) which requires the addition of a new IV or oral therapy for management. The event shall be considered resolved upon the discontinuation of the treatment.

Myocardial Infarction

Myocardial infarction will be identified if it is:

Peri-Operative Myocardial Infarction

The clinical suspicion of myocardial infarction together with CK-MB or Troponin > 10 times the local hospital upper limits of normal, found within 7 days following VAD implant together with ECG findings consistent with acute myocardial infarction. (This definition uses the higher suggested limit for serum markers due to apical coring at the time of VAD placement and does not use wall motion changes because the apical sewing ring inherently creates new wall motion abnormalities.)

or

Non-Perioperative Myocardial Infarction

The presence at > 7 days post-implant of two of the following three criteria:

- Chest pain which is characteristic of myocardial ischemia,
- ECG with a pattern or changes consistent with a myocardial infarction, and
- Troponin or CK (measured by standard clinical pathology/laboratory medicine methods) greater than the normal range for the local hospital with positive MB fraction (≥ 3% total CK). This should be accompanied by a new regional LV or RV wall motion abnormality on a myocardial imaging study.

Psychiatric Episode

Disturbance in thinking, emotion or behavior that causes substantial impairment in functioning or marked subjective distress and requires intervention. Intervention is the addition of new psychiatric medication, hospitalization, or referral to a mental health professional for treatment. Suicide is included in this definition.

Renal Dysfunction

Two categories of renal dysfunction will be identified:

Acute Renal Dysfunction

Abnormal kidney function requiring dialysis (including hemofiltration) in patients who did not require this procedure prior to implant, or a rise in serum creatinine of greater than 3 times baseline or greater than 5 mg/dL (in children, creatinine greater than 3 times upper limit of normal for age) sustained for over 48 hours.

Chronic Renal Dysfunction

An increase in serum creatinine of 2 mg/dl or greater above baseline, or requirement for hemodialysis sustained for at least 90 days.

Respiratory Failure

Respiratory failure is collected for episodes of re-intubation that occur after initial extubation, except for the situation where intubation occurs in the context of a diagnostic or brief therapeutic procedure.

Right Heart Failure

Symptoms or findings of persistent right ventricular failure characterized by **both** of the following:

- Documentation of elevated central venous pressure (CVP) by:
  - Direct measurement (e.g., right heart catheterization) with evidence of a central venous pressure (CVP) or right atrial pressure (RAP) > 16 mmHg.

    or
  - Findings of significantly dilated inferior vena cava with absence of inspiratory variation by echocardiography,

    or
  - Clinical findings of elevated jugular venous distension at least halfway up the neck in an upright patient.

- Manifestations of elevated central venous pressure characterized by:
  - Clinical findings of peripheral edema ($\geq$2+ either new or unresolved),

    or
  - Presence of ascites or palpable hepatomegaly on physical examination (unmistakable abdominal contour) or by diagnostic imaging,

    or
  - Laboratory evidence of worsening hepatic (total bilirubin > 2.0 mg/dl) or renal dysfunction (creatinine > 2.0 mg/dl).

Arterial Non-CNS Thromboembolism

An acute systemic arterial perfusion deficit in any non-cerebrovascular organ system due to thromboembolism confirmed by one or more of the following:

1. standard clinical and laboratory testing
2. operative findings
3. autopsy findings

This definition excludes neurological events.

Venous Thromboembolism

Evidence of venous thromboembolic event (e.g. deep vein thrombosis, pulmonary embolism) by standard clinical and laboratory testing.

Wound Dehiscence

Disruption of the apposed surfaces of a surgical incision, excluding infectious etiology, and requiring surgical repair.

Other event causing clinically relevant changes in health

An event that causes clinically relevant changes in the patient's health (e.g. cancer).

Other – Serious respiratory illness not requiring intubation
Other – Limb ischemia
Other – Limb necrosis
Other – Compartment syndrome
Other – Peripheral vascular injury
Other – Peripheral nerve injury
