# Supplementary figures and images for: Multicenter outcomes for ventricular assist device support for failed stage II palliation
Source: JHLT Open. 2023 Nov 17;3:100015. doi: 10.1016/j.jhlto.2023.100015 (PMC11935379; doi:10.1016/j.jhlto.2023.100015)

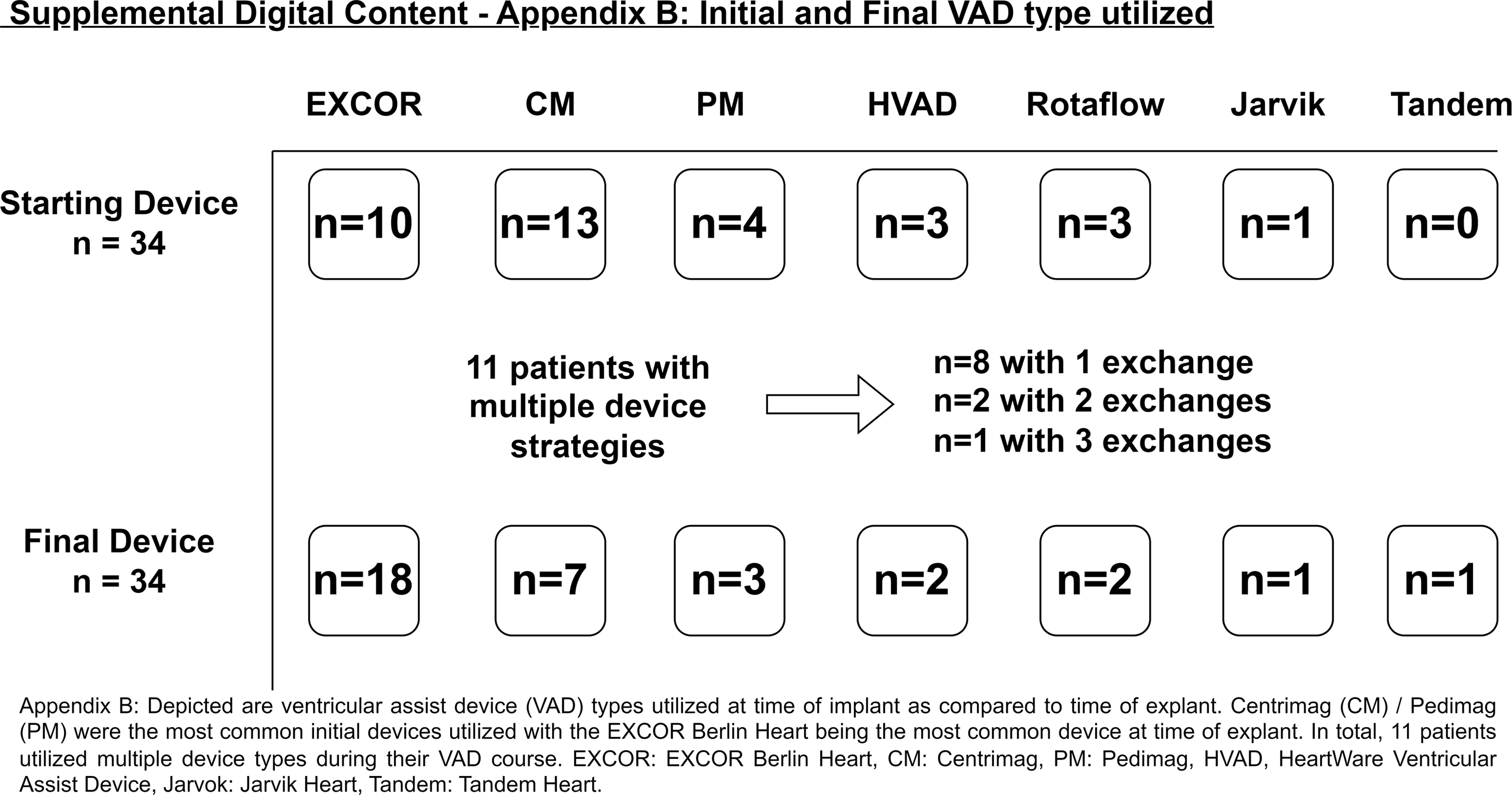

Supplement: Supplementary file 2 — Supplementary material [file mmc2.jpg]
